# Preventing Oral Dual Biofilm Development with Innovative Bioactive Varnishes

**DOI:** 10.3390/jfb16020070

**Published:** 2025-02-18

**Authors:** Tainá de Lima Costa, Regina Maria Puppin-Rontani, Aline Rogéria Freire de Castilho

**Affiliations:** 1 Departamento de Ciências da Saúde e Odontologia Infantil, Faculdade de Odontologia de Piracicaba, Universidade Estadual de Campinas, Piracicaba 13414-903, Brazil; tai-na@hotmail.com (T.d.L.C.); rmpuppin@unicamp.br (R.M.P.-R.); 2 Departamento de Odontologia Restauradora, Faculdade de Odontologia de Piracicaba, Universidade Estadual de Campinas, Piracicaba 13414-903, Brazil; 3 Department of Pediatric Dentistry, Indiana University School of Dentistry, Indianapolis, IN 46202, USA

**Keywords:** *Streptococcus mutans*, *Candida albicans*, dental caries, biofilms, natural products, flavonoids, terpenes, xanthines

## Abstract

This study introduces innovative varnishes incorporating natural bioactive compounds to inhibit the formation of oral dual biofilms, a critical contributor to dental caries and other oral diseases. The purpose of this study was to evaluate the effectiveness of bioactive varnishes containing *tt*-farnesol, quercetin, and theobromine in inhibiting the formation of mixed *Streptococcus mutans* and *Candida albicans* biofilms. Mixed biofilms of *Streptococcus mutans* UA159 and *Candida albicans* SC5314 were grown in 96-well plates containing a specialized culture medium. Approximately 0.2 mL of experimental varnishes with A—1.5% or B—4.5% concentrations of *tt*-farnesol, quercetin, and theobromine were separately added to the wells using a disposable applicator, with a vehicle varnish (lacking bioactives) serving as the control. Biofilms were incubated at 37 °C with 5% CO_2_ for 24 h. Microbial viability was determined in terms of colony-forming units per milliliter (CFU/mL), and biofilm morphology was evaluated qualitatively via scanning electron microscopy (SEM). Statistical analyses were performed using ANOVA/Tukey tests at a 5% significance level. Varnishes A and B achieved significant reductions in microbial populations within the biofilms (*p* < 0.05) compared to the vehicle control (C). SEM imaging revealed marked structural disruptions in the biofilms, validating the quantitative results. Higher bioactive concentrations demonstrated enhanced inhibitory effects. Bioactive varnishes enriched with theobromine, quercetin, and *tt*-farnesol represent a novel and effective strategy for inhibiting oral dual biofilm development, offering a promising advancement in preventive dentistry.

## 1. Introduction

Dental caries is a global health concern, representing the most widespread chronic disease affecting teeth [1]. This condition arises from a dysbiosis between the dental biofilm and the tooth surface, leading to the demineralization of hard tissues [2].

Knowingly, dental biofilm is an aggregation of microorganisms firmly attached to the tooth surface [3]. Among these microorganisms, *Streptococcus mutans* plays a central role in the caries process, being responsible for adhesion to the tooth surface and initiating biofilm formation [3]. However, *S. mutans* does not act in isolation [4,5]. Other species, such as the yeast *Candida albicans*, are involved in the microbial succession and often associate with *S. mutans*, enhancing the biofilm’s pathogenicity through their synergistic interaction [4,5,6,7]. While *S. mutans* is one of the primary colonizers of dental biofilm, facilitating the adhesion of new microbial cells via adhesins on its surface membrane [6], *C. albicans* has a high acidogenic potential that influences the biofilm’s biomass in the presence of fermentable sugars [8]. Although *C. albicans* does not typically promote the demineralization of dental hard tissues under normal conditions, a disruption in microbial homeostasis can lead to a synergistic interaction between *S. mutans* and *C. albicans*, increasing biofilm acidogenicity. This results in a decrease in pH and enhanced lactic acid production [6,7]. In other words, this cross-kingdom collaboration promotes increased biofilm formation, heightened acid production, and greater enamel demineralization, thereby contributing to the progression of dental caries [5,6,7,8]. Understanding the mechanisms underlying this interaction is crucial for developing effective and multi-target therapeutic strategies against cariogenic species [7]. Clearly, the presence of *C. albicans* amplifies the virulence of the cariogenic biofilm [3], highlighting the need for novel antimicrobial therapies to control biofilm formation and arrest carious lesions.

Bioactive compounds derived from natural products present a compelling opportunity to discover new therapeutic agents [9]. These substances have been widely explored in the pharmaceutical industry for their strong antimicrobial properties and established safety profiles [9,10,11].

Among natural bioactive compounds, the terpene trans–trans-farnesol (*tt*-farnesol) has demonstrated antimicrobial activity against planktonic *S. mutans* cells, as well as its ability to reduce the formation of *S. mutans* biofilm and extracellular polymeric substances [12,13,14,15].

Similarly, flavonoids are secondary metabolites found in plants that exhibit multiple pharmacological properties, including antioxidant and antimicrobial activities against pathogenic fungi, viruses, and bacteria [13,14,15]. These effects can result in growth inhibition, cell death, or enzymatic disruption, all while maintaining low toxicity to animal cells [16,17,18]. One of the most studied and widely recognized flavonoids is quercetin, known for its antioxidant and anti-inflammatory activities, as well as its antiviral, anticarcinogenic, psychostimulant, cardioprotective, and neuroprotective properties [19,20,21,22]. In dentistry, quercetin has demonstrated the ability to modulate the acidogenicity of *Streptococcus mutans* and promote dentin biomodification [23,24,25]. These properties highlight its potential to enhance dental treatments by reducing cariogenic activity and improving dentin structure [24].

Theobromine, an alkaloid found in cocoa beans, demonstrates significant anti-caries properties [26,27,28]. It enhances the remineralization potential of dental hard tissues, especially when combined with fluoride and calcium in formulations [26,27]. Furthermore, theobromine has been shown to reduce hydroxyapatite dissolution and form protective precipitates on the enamel surface during the caries process, contributing to its preventive role against dental caries [29].

Dental varnish is widely recognized as an effective professional fluoride treatment due to its prolonged adherence to the tooth surface, allowing for a sustained and controlled release of fluoride ions [30]. However, while fluoride is essential for caries prevention, its ability to modulate cariogenic biofilm composition and growth is limited [30]. To enhance its efficacy, incorporating natural bioactive compounds with antimicrobial and remineralizing properties into dental varnishes presents a promising alternative. These natural products have the potential to not only inhibit biofilm formation but also provide additional therapeutic benefits, making them a valuable advancement in preventive dentistry. Given the importance of controlling biofilms that lead to demineralization and contribute to the development of incipient caries, the development and characterization of novel therapeutic formulations containing natural products, such as dental varnishes, present a promising approach in dentistry, as these preventive materials may have the potential to disrupt oral biofilms. Additionally, factors such as the cost of commercial formulations and their clinical applicability in both public and private dental practices are critical considerations for their adoption as new therapeutic options.

Since the antibiofilm activity of the bioactive compounds *tt*-farnesol, quercetin, and theobromine has not yet been fully established, this in vitro study aimed to evaluate the inhibitory effects of bioactive varnishes containing these natural compounds on the formation of oral dual biofilms composed of *Streptococcus mutans* and *Candida albicans*. Taking the pharmacological properties of *tt*-farnesol, quercetin, and theobromine together, it is expected that those natural compounds could contribute to biofilm prevention through distinct yet complementary mechanisms. Specifically, this study sought to determine whether these varnishes could effectively reduce microbial viability and biofilm structural integrity. The hypothesis was that varnishes containing natural bioactives would significantly inhibit the formation of dual-species biofilms, demonstrating their potential as effective therapeutic agents for caries prevention.

## 2. Materials and Methods

### 2.1. Characterization of Formulations and Natural Compounds

Three different compounds were chosen to characterize the varnish formulations: *tt*-farnesol (C_15_H_26_O), quercetin (C_15_H_10_O_7_), and theobromine (C_7_H_8_N_4_O_2_) (Sigma-Aldrich, Steinheim, Germany). These compounds were used in two formulations (A and B) with different concentrations (A—1.5%; B—4.5%). The varnish without compounds (C) was used as the material control (C—0%). The details of the varnish preparation are described in the patent registry under National Invention Application, process BR 10 2020 021170 6, 2020.

### 2.2. Microorganisms, Culture Conditions, and Inoculum Preparation

**Figure 1:**
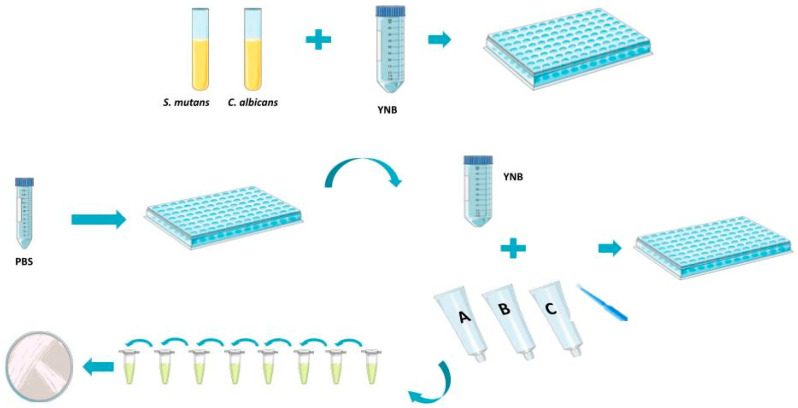
Schematic diagram of the assays performed in this study.

The microorganisms used in the experiments were *Streptococcus mutans* ATCC™ UA159 and *Candida albicans* SC5314. The reference strains were acquired from the Oswaldo Cruz Foundation (FIOCRUZ) collection and stored at −80 °C for preservation. *S. mutans* was grown in the BHI culture medium (Brain Heart Infusion, Difco Co., Detroit, MI, USA) at 5% CO_2_ and 37 °C for 18 h. The initial suspension was adjusted to an absorbance range of 0.08–0.1 at 625 nm (1.5 × 10^8^ CFU/mL concentration). *C. albicans* was grown in the Yeast Nitrogen Base (YNB; Difco Co., Detroit, MI, USA) culture medium at 5% CO_2_ and 37 °C for 24 h, and after the incubation period, the density was adjusted to 0.2 at 620 nm. Both microorganisms were incubated under static conditions to allow for biofilm formation for 24 h, and the biofilm density was assessed at regular intervals. Figure 1 represents a schematic diagram of the assays.

### 2.3. Antimicrobial Activity of Varnishes in Mature Mixed Biofilm

After adjusting the optical density of the *S. mutans* UA159 and *C. albicans* SC5314 samples, mixed biofilms were formed in a 96-well microtiter plate (Costar Corp., Cambridge, MA, USA using 100 µL aliquots of standardized cell suspension (1 × 10^8^ cells/mL for *S. mutans* and 1 × 10^7^ cells/mL for *C. albicans*) in 100 µL of the YNB culture medium (Difco Co., Detroit, MI, USA). The microorganism concentration mimics a high-risk caries situation where *S. mutans*, when coupled with *C. albicans*, can create an environment conducive for caries formation. The plates were then incubated statically at 5% CO_2_ and 37 °C for 24 h to promote biofilm formation.

Each well was washed once with 200 µL of phosphate-buffered saline solution (PBS; Gibco, Grand Island, NJ, USA; pH 7.4), to remove non-adherent cells, followed by the addition of 200 µL of the YNB culture medium (Difco Co., Detroit, MI, USA). Approximately 0.2 mL of each varnish (A, B, or C) was then separately added to the wells using a disposable applicator (microbrush), and the plates were incubated at 5% CO_2_ and 37 °C for 24 h. As a positive control of the experiments, 0.2% chlorhexidine digluconate (Sigma-Aldrich, Steinheim, Germany) was used, and wells inoculated with the culture medium only served as the negative control.

After incubation, each well was washed once with 200 µL of PBS to remove non-adherent cells. The PBS was removed, and 100 µL of fresh PBS was added to the wells. From each well, 10 µL aliquots were collected and transferred to Eppendorf tubes containing 90 µL of PBS. These aliquots were then diluted in decimal series from 10^−1^ to 10^−8^.

Finally, 10 µL of each dilution was inoculated in triplicate on BHI agar plates. Colonies were counted, and the number of viable bacteria was determined in terms of colony-forming units (CFU/mL of biofilm). All tests were performed independently and in triplicate.

### 2.4. Morphological Analysis of Biofilm in Scanning Electron Microscopy (SEM)

The mixed biofilms were grown on glass slides inserted in the microtiter plate (Costar Corp., Cambridge, MA, USA) and treated once with approximately 0.2 mL of each varnish (A, B or C), as described previously (Section 2.2 and Section 2.3). After 24 h of incubation, the slides were then washed with PBS to remove non-adherent cells and maintained for 2 h in 2.5% glutaraldehyde/PBS at room temperature. Afterward, the biofilm that adhered to the slides was dehydrated with ethanol (from 50% to 100%) and dried in a desiccator. The biofilm samples were then metallized (40 mA metallizer—BAL-TEC SCD 050) and observed under a microscope (Jeol JSM 5600LV, Tokyo, Japan) at 500× and 1000× magnification.

### 2.5. Statistical Analysis

An exploratory and descriptive analysis of the data was performed to assess whether they met the assumptions for parametric analysis. The errors were required to follow a normal distribution, be independent, and exhibit constant variance (homoscedasticity), and the model had to be additive.

The log-transformed data of colony forming units per mL of biofilm (CFU/mL) were analyzed using one-way analysis of variance (ANOVA), with Tukey’s test employed for multiple comparisons. All analyses were conducted using GraphPad Prism 9.2.0, with a significance level set at 5%.

## 3. Results

**Figure 2:**
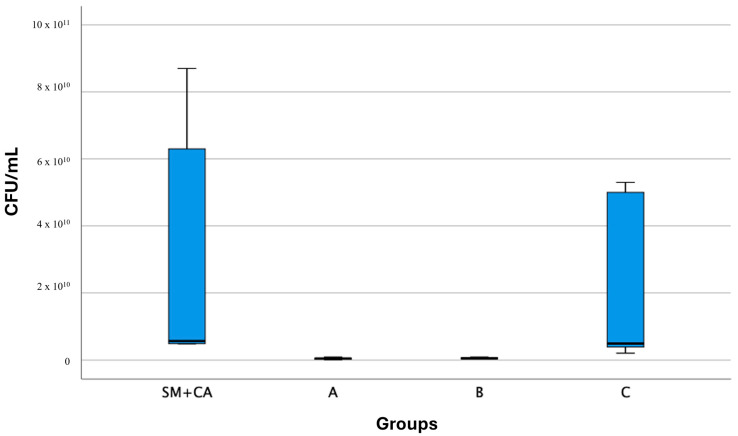
Box plot (mean and standard deviation) of the different varnish formulations (A, B, and C—control) for *S. mutans* (SM) and *C. albicans* (CA) biofilms (CFU/mL) (*n* = 6).

The representative data of dual biofilm inhibition showed a significant decrease in viable cells after the treatment with varnish formulations A (6.11 ± 0.32) and B (5.84 ± 0.14) but no significant change after the treatment with C (6.77 ± 0.73) (Table 1; Figure 2). Therefore, the biofilm of *S. mutans* and *C. albicans* (SM + CA) was susceptible to experimental formulations A and B (*p* < 0.05, ANOVA/Tukey), specially B, which differed statistically from the other groups (SM + CA and control C).

Figure 2 shows a higher number of colony-forming units (CFU) in the SM + CA group (7.09 ± 0.65) compared to groups A and B (*p* < 0.05). Additionally, there was a greater number of CFU in group C than in group B (*p* < 0.05).

**Figure 3:**
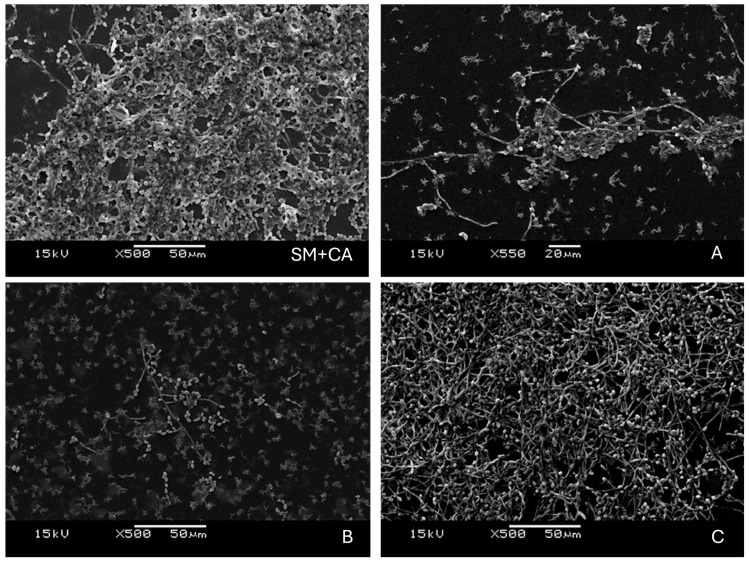
Scanning electron microscopy of *S. mutans* and *C. albicans* biofilms. Images show the typical architecture of a dual biofilm (SM + CA), with fewer cells after treatment with different concentrations (**A**,**B**) and no biofilm reduction ((**C**)-vehicle) at 1000× magnification. Reprinted from Ref. [31].

The qualitative verification of biofilm morphology using scanning electron microscopy revealed structural and numerical changes in the biofilm population among groups A, B, and C, as well as in the SM + CA microorganisms. Biofilms treated with varnishes A and B exhibited a significant reduction in total biomass compared to biofilms grown without bioactives and those treated with the control formulation (SM + CA and C) (Figure 3).

## 4. Discussion

Biofilms exhibit greater resistance and physical–chemical tolerance to antimicrobial agents compared to planktonic cells due to the complexity of the extracellular polysaccharide matrix that surrounds them, forming a protective barrier [5,32,33]. The oral dual biofilm model used in this study, which combines *S. mutans* and *C. albicans*, mimics the clinical scenario and provides insights into the complexities of biofilm formation in a mixed-species environment. The main finding of this study is that bioactive varnishes containing *tt*-farnesol, quercetin, and theobromine significantly inhibited the formation of dual-species biofilms composed of *S. mutans* and *C. albicans.* This was evidenced by a reduction in microbial viability and structural disruptions observed in the biofilms, confirming the hypothesis that natural product-based varnishes can effectively inhibit the formation of oral dual biofilms and demonstrating their potential as therapeutic agents. Although this is the first time that *tt*-farnesol, quercetin, and theobromine have been proven to exhibit antimicrobial activity when combined in a formulation, numerous studies have already strongly established their individual potency in inhibiting biofilm formation [34,35,36].

When considering the biofilm formed through the association of *S. mutans* and *C. albicans*, it is essential to highlight the increase in extracellular polysaccharides [33], particularly the insoluble polysaccharides, which are key virulence factors in cariogenic biofilms [4,37]. In the presence of *C. albicans*, *S. mutans*’s glycosyltransferase B strongly binds to the fungal surface, stimulating the production of a glucan-rich matrix [33]. This matrix is commonly observed in destructive caries lesions, indicating that the close association between these species is a determinant factor in the formation of a more virulent cariogenic biofilm [3,5]. Although this study did not observe an inhibition of polysaccharides by the varnishes, the formulations A and B significantly reduced the viability of microorganisms, clearly demonstrating an inhibitory effect on mature biofilms. Therefore, bioactive varnishes could be a promising adjunctive therapy for biofilm-related oral diseases, especially in high-risk populations, such as immunocompromised individuals, who are more vulnerable to opportunistic infections and severe caries progression [38,39,40]. Immunocompromised patients, including those undergoing chemotherapy, organ transplant recipients, and individuals with uncontrolled diabetes, often experience an imbalance in their oral microbiota, leading to increased colonization by pathogens like *C. albicans* [38,39].

*Candida albicans* exhibits fungal dimorphism, a property that enables morphological changes between yeast and hyphal forms, enhancing its pathogenesis and persistence in the oral cavity, as well as its cohesion to other oral bacteria [41]. Previous studies have shown that when hydroxyapatite serves as a substrate in mixed biofilms, *C. albicans* and *S. mutans* aggregate, demonstrating a high affinity between the cariogenic bacteria and the hyphal form of the fungus [3]. In the current study, scanning electron microscopy revealed that treatment with experimental varnishes affected microbial morphology, decreasing the number of microorganisms and altering the biofilm structure. Varnishes A and B led to a less organized biofilm with more porous and less intertwined cells compared to the more structured biofilm observed in the control group. Furthermore, fewer hyphae of *C. albicans* were present in biofilms treated with varnishes A and B, suggesting that these treatments modulate biofilm structure, reducing the pathogenicity of *C. albicans* and controlling the acidogenicity of * S. mutans*. Considering the relevance of this finding, an indirect treatment approach using the varnish in oral biofilms may offer benefits in managing invasive candidiasis by inhibiting Candida biofilm formation in the oral cavity, potentially reducing the oral reservoir of Candida and helping to prevent further systemic dissemination [39]. Additionally, this approach could be beneficial for preventing bacterial endocarditis, as *S. mutans*, a key pathogen in endocarditis, can form biofilms in the oral cavity and may enter the bloodstream, especially in individuals with compromised immunity [40]. However, a careful consideration of its potential side effects is necessary in immunocompromised patients. Monitoring the impact on the oral microbiome and assessing the risk of opportunistic infections are essential steps in evaluating the safety and efficacy of such treatments.

In this study, a single-dose therapy with the experimental varnishes was used. Therefore, it can be assumed that the inhibitory effect of the formulations could be enhanced if the varnishes were applied to the tooth surface multiple times over consecutive days or weeks, following clinical protocols for dental varnish application [42]. The regular use of the varnishes as a preventive dental material would provide a gradual and continuous release of the compounds in biofilms, early caries lesions, and saliva. In general, the current recommendation for treating oral candidiasis involves the topical use of antifungals 3 to 4 times a day for 2 to 4 weeks [43]. Thus, the pathogenesis of *C. albicans* in mixed biofilm formation would likely be more effectively modulated with more frequent use of the bioactive varnishes. While more frequent application is anticipated, extrapolating to a real-world scenario, the varnish represents a promising alternative for combating fungal and bacterial resistance. The varnishes studied here contain natural compounds, some of which are recommended as dietary supplements, and effectively targets both bacterial and Candida cells while maintaining concentrations that would minimize the risk of systemic toxicity. However, further studies are essential to fully assess their long-term efficacy and safety in clinical settings.

In a similar study, a varnish containing Brazilian green propolis extract was able to control the growth of cariogenic species *S. mutans*, *S. sanguinis*, *S. salivarius*, and *L. casei* [44]. As observed in this study, varnish inhibited the growth of a multispecies biofilm, which is more resistant than planktonic cells and monospecies biofilms. Another recent study analyzed the antimicrobial and anti-caries effects of a TiF4 varnish on microcosm biofilms formed on enamel [45]. Although the TiF4 varnish reduced enamel demineralization, it was not effective in interfering with the adhesion of microorganisms, growth, and biofilm activity [45], possibly due to the limited antimicrobial activity of fluoride.

When comparing the antimicrobial effects of titanium tetrafluoride (TiF4), chlorhexidine (CHX), xylitol, and sodium fluoride (NaF) on *S. mutans*, TiF4, CHX, and NaF were effective against *S. mutans*, while xylitol was unable to cause a significant inhibitory effect at any concentration [46]. Furthermore, TiF4 was not capable of decreasing microbial cell viability up to a 6.25% concentration, as evidenced by the minimum inhibitory concentration (12.5%) and the minimum bactericidal concentration (25%), making it unfeasible for application in the oral cavity. It is important to note that Eskandarian et al., 2017 [46] used a planktonic model, which is a microbiological model typically used for initial studies. This model is controlled and more susceptible to antimicrobial agents due to the absence of the polysaccharide matrix that protects microorganisms in biofilms. Therefore, this model was unable to simulate the real conditions in the oral cavity. Even when analyzing the effect of the TiF4 varnish in situ, no inhibitory properties were detected against total streptococci, *S. mutans*, Lactobacillus, or *Candida* spp. after 15 days of biofilm formation. While studies have focused on fluoride’s efficacy, it is widely considered the gold standard for controlling and preventing caries due to its role in the demineralization and remineralization processes. However, as an antimicrobial agent, fluoride is not as effective as it does not fully control the infectious process of the disease on its own. Chlorhexidine, another widely used antimicrobial agent, is indeed effective against *S. mutans* and other oral microorganisms. However, its long-term use has been restricted due to side effects such as tooth staining, an unpleasant taste, and the suppression of the regular oral microbiota, which can allow more virulent species to colonize the tooth surface [46]. Despite its broad-spectrum activity, CHX’s limited effectiveness in disrupting biofilms has prompted the search for more sustainable and cost-effective alternatives.

Varnishes represent an innovative approach in dental care, offering several advantages due to their alcohol-based formulation, which ensures fast drying and strong adhesion to the tooth surface [30,42]. This facilitates a controlled, prolonged release of bioactive compounds, maintaining therapeutic effects for hours and enhancing the efficacy of preventive treatments while reducing patient dependence on compliance [30]. The natural compounds incorporated into these varnishes, such as quercetin, *tt*-farnesol, and theobromine, provide additional benefits. Quercetin has demonstrated antibiofilm activity against *S. mutans*, reducing dry weight, proteins, viable cells, and soluble and insoluble glucans and even lowering the pH of biofilms [47]. Similarly, *tt*-farnesol has shown a significant reduction in extracellular polysaccharides in mixed biofilms of S. mutans and *C. albicans*, highlighting its potential to disrupt a key virulence factor of these biofilms [13]. Theobromine, derived from cocoa, exhibits remineralizing properties, forming hydroxyapatite crystals that reinforce enamel structure [29].

In fact, the combination of different natural compounds offers a more complex formulation capable of addressing multiple therapeutic targets. Considering daily clinical practice, the experimental varnish has the potential to demonstrate dual action as both an antimicrobial agent and an anticariogenic treatment. Its ability to inhibit biofilm formation provides an alternative approach to biofilm control, effectively targeting cariogenic and opportunistic pathogens while mitigating concerns related to microbial resistance. Additionally, the presence of theobromine in its composition suggests a possible remineralizing effect, which could contribute to enamel strengthening and the repair of early carious lesions. This combined potential positions the varnish as a promising preventive strategy; however, further well-designed studies are necessary to elucidate its anticariogenic properties and establish long-term clinical efficacy.

The findings in this current study suggest that the varnish may have a significant impact on inhibiting biofilm formation, particularly against cariogenic and opportunistic pathogens such as *S. mutans* and *C. albicans*. However, several limitations should be acknowledged when interpreting these results, which could influence the estimation of the varnish’s true potential. One of the most important limitations of this study is the use of a single-dose application of the varnish. In real-world clinical settings, regular varnish applications would allow for the continuous release of bioactive compounds, thereby enhancing therapeutic effects and providing prolonged protection against biofilm formation. Therefore, the single-dose application in this study may underestimate the therapeutic effect of the varnish, and studies with repeated applications would be necessary for a more comprehensive evaluation of its effectiveness. Additionally, the short duration of the study did not allow for an assessment of the varnish’s long-term impact, which is crucial for determining its clinical efficacy in real-world scenarios. Long-term follow-up would provide data on the persistence of antimicrobial effects, as well as the varnish’s ability to promote remineralization over time. The absence of clinical data on long-term outcomes may lead to an overestimation of the immediate effects observed, without a detailed analysis of their maintenance or potential cumulative effect. Another important point is the varnish composition, which includes natural compounds such as quercetin, *tt*-farnesol, and theobromine. Together, these compounds have shown antimicrobial properties, which are promising for controlling caries and oral fungal infections, such as candidiasis and remineralizing potential, which could contribute to the repair of incipient caries lesions. Thus, the biochemical property warrants further investigation, given its potential benefit in preventive dentistry. In terms of antimicrobial resistance, the use of natural compounds offers a promising alternative to conventional treatments, opening up new possibilities for innovation in dental care, especially in an era of growing resistance. However, the varnish’s potential to prevent the development of microbial resistance remains unknown.

In summary, given the complexity and resilience of biofilms formed by *S. mutans* and *C. albicans* and considering the limitations of this study, the findings emphasize the significant potential of bioactive varnishes containing natural compounds as an effective antibiofilm therapy. These varnishes represent an innovative, sustainable, and cost-effective alternative for controlling biofilm formation, positioning them as a promising solution for caries prevention.

## 5. Conclusions

Despite the limitations of this in vitro study, it can be concluded that the bioactive varnish containing natural products demonstrates a significant inhibitory effect on oral dual biofilms and could be an alternative for caries prevention.

## Figures and Tables

**Table 1:** Tukey’s multiple comparisons test results.

Comparison	Mean Diff	95% CI of Diff	Adjusted *p* Value
MO vs. A	0.9796	0.2710 to 1.688	0.004 **
MO vs. B	1.245	0.5362 to 1.953	0.0003 ***
MO vs. C	0.3178	−0.3908 to 1.026	0.6169
A vs. B	0.2652	−0.4434 to 0.9738	0.7382
A vs. C	−0.6618	−1.370 to 0.04680	0.0737
B vs. C	−0.927	−1.636 to −0.2184	0.0068 **

MO—microorganism (SM + CA; experiment control); A—1.5%; B—4.5%; C—without bioactive. Significant and highly significant differences are represented by (**) for *p*-values of less than 5%, and (***) for less than 1%, respectively.

## Data Availability

The original contributions presented in the study are included in the article, further inquiries can be directed to the corresponding author.
